# A Conceptual Analysis of the Semantic Use of Multiple Intelligences Theory and Implications for Teacher Education

**DOI:** 10.3389/fpsyg.2022.920851

**Published:** 2022-06-16

**Authors:** Adam I. Attwood

**Affiliations:** Department of Teaching and Learning, Austin Peay State University, Clarksville, TN, United States

**Keywords:** teaching of educational psychology, general intelligence, universal design for learning, understanding by design, multiple intelligences theory, preservice teachers, differentiation, semantics

## Abstract

Gardner’s theory of multiple intelligences (MI) has been at the center of a long-running debate in educational psychology in terms of its generalizable validity. In this article, MI theory is discussed for a review of why and how MI theory may be contextually discussed for preservice teachers to learn about in their teacher education program. The semantic conceptual basis of intelligence in MI theory is discussed in comparison to learning styles theory with implications for the importance of the teaching of Universal Design for Learning and related frameworks in teacher education curriculum.

## Introduction

Gardner’s (1983, 2006) theory of multiple intelligences [hereafter referred to as multiple intelligences (MI) theory] has had substantial influence on K-12 curriculum design and implementation. This influence has been promoted, at times, through professional development for in-service teachers and in teacher education programs for preservice teachers (see, for example, “Project Zero” from the Harvard Graduate School of Education; Multiple Intelligence Schools, 2022). The seven and then eight intelligences that comprise the theory that Gardner (1983, 2006) posited have influenced teachers, students, and teacher educators across the world (Brualdi Timmins, 1996; Rousseau, 2021). This influence has generated a long-standing debate among educational psychologists about the efficacy and validity of MI theory. While there has been substantial discussion of the varying points of view of educational psychologists and teachers on MI theory, there is not nearly as much discussion of this issue for teacher educators who address MI theory in what may be the only educational psychology course that preservice teachers take during their teacher education program. There should be more literature specifically on the teacher educator perspective on how to teach MI theory to preservice teachers in comparison to Understanding by Design (UbD) and Universal Design for Learning (UDL). This paper reevaluates the debate about the efficacy and validity of MI theory, and makes recommendations for how this debate can be introduced and discussed in a survey course on educational psychology for preservice teachers.

## Rationale

Most preservice teachers seem to have a favorable opinion of Gardner’s MI theory (Rousseau, 2021). This is despite the long-standing critical debate on the theory’s efficacy and validity from the technical perspectives of educational psychologists (Schulte et al., 2004; Bordelon and Banbury, 2005; Visser et al., 2006; Waterhouse, 2006; McGreal, 2013; Rogowsky et al., 2015; Willingham et al., 2015; Rousseau, 2021). Additionally, a study by Luo and Huang (2019) of English as a second language (ESL) teachers’ self-perception of MI theory and the uses of the defined multiple intelligences found either ambiguity or no significant correlation between MI theory and its instructional strategies, further supporting the critics of MI theory based on it not having statistical validity. These findings prompt the question of why? Why do preservice teachers tend to have an overall positive opinion of MI theory? This paper addresses these questions as a conceptual issue in semantics.

Part of the answer for preservice teacher perception of MI theory may be in the perspectives they developed in either their teacher education program or in their school placement (Rousseau, 2021). This may present as having heard about anecdotal success in applying MI theory, and then assuming it is beneficial in and of itself without critical evaluation of whether theories such as MI theory are supported in psychological science. The other part of the answer is in the educational psychological research that supports MI theory in qualitative principle but not in the technical aspects of statistical validity. These can become conflated when preservice teachers go into their field placements and hear the words *differentiation* and *learning styles* and semantically linking these to *preferences* in an unquestioning assumption because of perceived popularity despite technical differences among these concepts in psychological science, especially in reference to the word *intelligence* in context of MI theory. This second reason reinforces the first because preservice teachers’ perception of the effectiveness of learning styles and related theories, especially MI theory, tends to be influenced by anecdotal experience (Menz et al., 2021).

## Comparative Literature Review

### Interpreting the Research Links and Conceptual Overlap of MI Theory and Learning Styles Theory

While most of the critique of MI theory is in the technical use of the word “intelligences” and the testing of MI theory’s validity in correlating the intelligences to teaching and learning that have found no correlation between MI theory and its instructional strategies (e.g., Schulte et al., 2004; Visser et al., 2006; Waterhouse, 2006; Rogowsky et al., 2015; Willingham et al., 2015; Luo and Huang, 2019), there are a number of studies that demonstrated favorable findings that partially validate MI theory, though there are qualifying limitations (e.g., Mokhtar et al., 2008; Furnham, 2009; Wu and Alrabah, 2009; Dolati and Tahriri, 2017; Prast et al., 2018; Yidana et al., 2022). However, Pashler et al. (2008) suggested that some studies that suggest favorable findings of learning styles theories there might be ambiguity in the study design leading to inconclusive findings. The issue, again, seems to be predicated on the semantics of MI theory in its use of the word intelligences. Other studies have emphasized contextual interpretation with statistical data analysis involving in-service teachers or preservice teachers to provide evidence of MI theory’s qualitative effectiveness in the multiple-subject curriculum of elementary school to secondary school science and mathematics (e.g., Baş and Beyhan, 2010; Modirkhamene and Azhiri, 2012; Milić and Simeunović, 2017; Koçak Altundağ, 2018; Ghaznavi et al., 2021; Shahzada et al., 2021). These case studies further support the efficacy of MI theory in classroom practice.

Armstrong’s (2018) textbook *Multiple Intelligences in the Classroom* (in its fourth edition in 2018) demonstrates what seems to continue to be perennial interest—and, perhaps it could be said, popularity among teachers—of MI theory for inspiring engagement with ways in which to differentiate instruction and link with the related learning styles theories. Multiple intelligences theory and learning styles theory, while different, do interface with each other in that the theory of learning styles could be viewed as the conceptual framework while MI theory is an operationalization of that framework. According to Silver et al. (1997):

“Though both theories claim that dominant ideologies of intelligence inhibit our understanding of human differences, learning styles are concerned with differences in the *process* of learning, whereas multiple intelligences center on the *content* and *products* of learning. Until now, neither theory has had much to do with the other” (para. 2).

With MI theory’s continued place in popular discussion, it should be contextualized in educational psychology courses for preservice teachers with an emphasis on additional evidence-based frameworks. These evidence-based frameworks include “Understanding by Design” (UbD; see Wiggins and McTighe, 2005) and UDL (see Hall et al., 2012).

#### Role of Anecdotal Evidence

Psychology is sometimes viewed as a helping profession as it is often applied across human services fields (Sternberg and Dennis, 1997). Teacher education is historically rooted in the field of psychology. As a social science, psychology’s use of validity tests of pedagogical strategies is important. That being stated, teaching—as a helping profession—is also as much about the teacher’s intuition informed by their teaching experience and their anecdotal observations of a lesson’s effects historically and in the present on student learning. Anecdotes matter because teaching can differ from textbook theories, even if those psychological theories have validity. As such, historical study and psychological study are linked, and this can often include individual stories (Vaughn-Blount et al., 2009). Anecdote should not replace psychological science, but neither should quantitative studies be used to the exclusion of all anecdotal experience as an important source of information about teaching practice. These are different constructs that each hold a place in educational psychology. The stories of anecdotal observation by Stock (1993) and Weber (1993), for example, are examples of the importance of anecdotes in assessing teaching and learning. Similarly, the importance of the anecdote and anecdotal method has been established for its helpful role in teacher professional knowledge construction (Doecke et al., 2000; Attwood, 2021).

Although the effect of anecdotal evidence can be substantially influential, the quality of anecdotes can be difficult for individuals to discern. Hornikx (2018) noted in their study testing the effect on participants’ opinions using “high-quality” and “low-quality” anecdotal evidence that “contrary to theoretical expectations, readers were not found to be sensitive to the quality of anecdotal evidence: The similar and dissimilar variants were equally persuasive” (p. 333). Such a finding might offer further basis for why MI theory has been found to be popular with preservice teachers when considering the findings of Rousseau (2021). It also might underscore some of the concerns of critics of MI theory who suggest that MI theory lacks sufficient scientific basis for its claims regarding multiple intelligences (Waterhouse, 2006; Willingham et al., 2015). The critique of validity in strategies such as MI theory is important and should be emphasized in educational psychology, but the qualitative and anecdotal observations of teachers who are daily in their classrooms seeing favorable results from differentiated practice is at least as important and should likewise have a place in educational psychology curriculum. Sometimes, the semantics of the framework becomes an obstacle and, instead, the observed results in the classroom might matter more. It is in that perspective, then, that MI theory continues to influence some educators in presenting MI theory with what appears to be an overall favorable impression of the theory’s possibilities.

Although there has been a substantial phase of popularity in the critique of “neuromyths” prompted, in part, by critical studies of MI theory during the 2000s and 2010s, this appears to be waning and perhaps partially reversing, at least temporarily (Gardner, 2020; Rousseau, 2021). An example of the popularity of critique has been in the widely assigned textbook *Educational Psychology: Developing Learners* by Ormrod et al. (2020), which by 2020 was in its tenth edition. This textbook for undergraduate preservice K-12 teachers and psychology students features a critique of “neuromyths” in the first chapter. Among the critiques is one focused on learning styles theories and Gardner’s (1983, 2006) MI theory. There was some ambivalence in the critique, but it was more in the negative.

Considering how widely used this textbook is in survey courses in educational psychology in the United States, this raises the earlier question, again: Why do preservice teachers still seem to have an overall positive opinion of MI theory, as Rousseau (2021) discussed in a review of several international studies. Part of the reason is what may be an emphasis on qualitative interpretation rather than quantitative inquiry in educational psychology courses for preservice K-12 teachers because they will be teaching in one school and informed by their own individual teaching experience and the experience of their supervising teacher to adapt to the conditions of their local classrooms in consultation with colleagues. In this scenario, the individual experience in full-time student teaching might take precedence, at least in the near term, over the studies that critiqued MI theory that they may have read previously.

#### Connecting Concepts in Learning Styles Theory Across Contexts of Language

It is important for teacher educators to make the connection between studies in psychological science and the daily routines of K-12 schools evident for preservice teachers. Achieving this connection in their educational psychology course is important for encouraging preservice teachers to emphasize evidence-based pedagogical frameworks (e.g., UbD and UDL) in their lesson planning and practice as classroom teachers. This connection should be established so preservice teachers may contextualize anecdotal experience with studies in psychological science to foster understanding of evidence-based frameworks, they might be less likely to continue to have misconceptions about MI theory and learning styles theory (Menz et al., 2021; Rousseau, 2021).

When teacher educators teach the importance of both qualitative and quantitative inquiry in educational psychology, preservice teachers can decide what will work for them in the classroom. Contextualizing technical scientific validity matters for how to translate those studies to what seems to work and can most efficiently be communicated for and in the K-12 classroom. Preservice teachers should be taught frameworks for implementing effective instruction and classroom management, and this can involve teaching about MI theory to get preservice teachers thinking about intentional, inclusive curriculum design. Thus, teacher educators should probably emphasize what MI theory has to offer practically to encourage effective differentiation of instruction.

There are increasingly more examples of demonstrating connections between research that tests MI theory and presents favorable findings of MI theory. A study of secondary school economics teachers found that there was a statistically significant difference using a multiple analysis of variance in teachers’ use of a bodily-kinesthetic approach to teaching economics in secondary school based on the teacher’s teaching experience (Yidana et al., 2022). In a study of English as a foreign language (EFL) teachers, the researchers found that “only teachers of logical-mathematical type were influenced by their dominant intelligence type and other intelligence types did not exert a significant influence on the types of activities being implemented in the classes” (Dolati and Tahriri, 2017, p. 1). It was also notable that the majority of the teachers in that study “did not have any education at the university level about the MI theory” (Dolati and Tahriri, 2017, p. 7). This indicates further support for the logical-mathematical intelligence construct in MI theory because the teachers in that study were not taught about MI theory in their teacher education program suggesting little to no prior influence on their opinion of the theory. In a study comparing EFL students internationally and applying MI theory in a cross-cultural analysis, Wu and Alrabah (2009) applied MI theory to identify students’ learning preferences to develop a differentiated teaching approach. These studies further suggest the popularity of MI theory for its pedagogical potential in successfully implementing a differentiated curriculum. The question, then, is prompted again to what the extent of overlap between MI theory’s concept of plural intelligences is and whether it is being used in practice as nearly synonymous with *preferences*. This is especially relevant when considering MI theory’s relationship to—and operationalization of—learning styles theory (Silver et al., 1997).

### Precision in Word Choice of Intelligences or Preferences at Center of MI Theory Debate

If the word *intelligences* were replaced with the word *preferences* in MI theory, there might be a shift in the debate of MI theory’s efficacy. The semantics of MI theory’s context in teacher education may be part of the reason why many preservice teachers may tend to have a favorable perception of MI theory because the concept of intelligences seems to be used synonymously with preferences. Nevertheless, a student’s *preference* in modality (i.e., auditory) may still not necessarily be the best approach for their learning depending on the topic, skill, and activity being taught, though there is some conflicting evidence (Rogowsky et al., 2015). Approaching MI theory from a classroom practitioner perspective is generally how preservice teachers will interact with the concept of MI theory. With this understanding, it makes sense that technical validity studies would be of less importance if the classroom observations of MI theory’s implementation appear to support its use for K-12 classroom instruction.

Differentiation is, in part, about aligning instruction to student preferences as much as anything else for the purpose of maintaining or increasing student engagement. This has been one of the benefits of MI theory in teacher education. The debate about MI theory has perhaps, ultimately, been more about the semantics of the word intelligences and observations of anecdotal benefits of its implementation. The technical aspects of quantitative validity are important, but not always to a level that supersedes the qualitative effectiveness of the theory’s application if the discussion is focused on preservice teachers’ observations of its use in classrooms. If students appear engaged in the content when using MI theory, then perhaps that makes more of a positive difference for in-service teachers. Even when validation tests do not meet or exceed the 95 percent confidence interval, there will still be anecdotal cases of apparent success in the theory’s implementation (e.g., Furnham, 2009).

#### Semantics of MI Theory Affect Assessment Conceptualization

Student engagement is often part of formative assessment in K-12 classrooms, hence part of the reason that MI theory became popular in the late twentieth century as a potential way to address differentiated engagement. This is where more recent discussion in the MI theory debate has trended. When teachers assess students based on MI theory or related, though different, learning styles theory in their classroom, Papadatou-Pastou et al. (2018) observed that there can be a dissonance in the *preferences* being assessed by the teacher and the students’ self-assessment of their own preferences on the given task. If using the MI theory as a lens, preferences can become conflated with the definition of intelligence used in MI theory. In a broader discussion of self-assessment, Coutinho et al. (2021) addressed the related issue of the Dunning-Kruger effect in the debate on learning styles theories when student preferences are emphasized to the point that they could potentially over-rely on self-assessment.

If the word intelligence is changed to preference, it seems that the issue of validity may not be as relevant, and self-assessments could be checked against the teacher’s assessment of the given task or assignment so that the unit grade is not based on the student’s self-assessment. The word change would also likely address Willingham et al. (2015) in their critique of learning styles theories and the inferred influence of Gardner’s (1983, 2006) MI theory. To avoid this semantic issue, preservice teachers would benefit more from being taught UDL which addresses student preferences within an evidence-based framework so that their learning is personalized to what will more likely generate effective learning instead of relying solely on a student’s self-reported preference which may not be accurate or best for their achievement of the learning goals.

When testing MI theory to ascertain if it holds validity in assessing instructional strategies aligned to its concept of multiple intelligences, many studies have found no widespread statistical correlation or validity in MI theory’s concepts of multiple intelligences and instructional strategies aligned to those purported intelligences (e.g., Waterhouse, 2006; Willingham et al., 2015; Luo and Huang, 2019). However, some case studies have found statistical significance for some of the MI domains (e.g., Dolati and Tahriri, 2017; Yidana et al., 2022), or partial significance for one but not the other domains (e.g., Rogowsky et al., 2015). While divergent findings further the debate of MI theory, this again seems to suggest the issue of the semantics of this theory and its overlap with learning styles theory, as the use of the word “intelligence” in MI theory can cause confusion from a technical standpoint in psychological science where the word *preferences* may be a better fit for Gardner’s theory, especially in context of its overlapping application with learning styles theory.

Some studies that emphasized qualitative observation in assessment practices and semantically infer that the use of the word intelligences is practically synonymous with preferences suggest more positive results, though not generalizable (e.g., Mokhtar et al., 2008; Baş and Beyhan, 2010). However, when testing MI theory’s application in how assessment of student learning is conducted in an MI theory-based curriculum, findings from studies suggest ambiguity or little scientific foundation for MI theory (Luo and Huang, 2019). This, taken together with other studies that raise questions of MI theory’s generalizable significance or lack of correlation between it and assessment strategies based upon it (Waterhouse, 2006; Willingham et al., 2015), provide additional support for explicit direct instruction (EDI) in the teaching of English as a second language, as well as in the teaching of several other content areas. EDI, itself, overlays with UDL as part of the highly structured and scaffolded approach in direct instruction (Hollingsworth and Ybarra, 2018).

#### Semantics of General Intelligence (*g*) or Intelligences Affect Student Engagement Conceptualization

The importance of teaching about MI theory from a student engagement perspective has been debated with some scholars suggesting that granular technicalities of quantitative validity studies in the learning styles theories are not always more important than the qualitative influence of such theories in improving instruction (Shearer, 2004). As suggested by some scholars, MI theory has benefits when implemented intentionally and systematically across learner groups from those below grade level to those above grade level (Milić and Simeunović, 2017; Shearer, 2020). Gardner (1983) essentially changed the meaning of the word intelligence in the way it was used in his book. According to Shearer (2004), “It is fundamentally important to recognize that MI [multiple intelligences theory] is a new kind of construct based on a unique definition of intelligence” (p. 3). Shearer (2004) criticized some of the earlier critics of MI theory for a “distorted understanding of the theory itself” (p. 2) and that some critics were misapplying the general intelligence (known as *g*) construct by using a different definition than the one Gardner (1983) was using.

The semantics are important as there should be clear definitions so that discussants are speaking from the same understanding of the words used. As Shearer (2004) argued, Gardner was not basing MI theory on the older, extant concept of a singular general intelligence (*g*) but, instead, Gardner was positing a new construct in which a singular general intelligence was deemphasized while positing the concept of MI when using the construct for K-12 teaching and learning. Nevertheless, there need not be a dichotomy between using MI theory and not using it. Instead, in teacher education, there should be nuance in its use in keeping with the complex context of schools with students below, at, and above grade level in the same classroom.

Fostering learning for all students to achieve the learning targets is central to lesson planning, and if MI theory’s implementation achieves that goal, then it is useful for preservice teachers to learn. This was inferred in a study that showed some positive findings of the intelligence domains outside of *g*, thus providing some tentative support for MI theory (Visser et al., 2006). Even small associations or even inconclusive associations of MI theory can still have qualitatively important outcomes for students. As such, the intelligence quotient (IQ)—influenced by the research on *g*—has been revised several times with subsequent research, suggesting that the IQ is important but not a static concept (Kaufman, 2018). One view is that transcending dichotomies of using or not using MI theory is necessary, and instead use what works from MI theory on a classroom-by-classroom basis which is part of what MI theory is focused on: choice based on preferences. This still becomes semantically complicated because of what definition of “intelligences” is being used. However, UbD and UDL offer more useful frameworks without the semantic debate. UbD and UDL should be emphasized in educational psychology courses as they will serve as evidence-based frameworks for preservice teachers to learn about lesson planning that is more likely to be effective. UDL has been substantiated in research studies, including a study using an item-level content validity index (I-CVI) that demonstrated how a UDL-based approach in grades 6–12 supported “personalized learning” (Zhang et al., 2022).

## Discussion of Teaching Implications

MI theory provides an entry point for discussion of differentiation. However, UbD and UDL should be emphasized and taught in educational psychology for preservice teachers so that these strategies are accessible to demonstrate their usefulness across school contexts in successfully implementing differentiated strategies across content areas and grade levels. Providing preservice teachers with examples is essential. Assigning a lesson plan project in which preservice teachers apply UDL, for example, will help establish understanding and confidence to use this evidence-based approach. In a study of preservice teachers who were taught the UDL framework for lesson planning, Spooner et al. (2007) found that preservice teachers in both special education and general education benefited substantially from just one lesson on UDL. With instruction in UDL with practice, preservice teachers could gain even more benefit in this evidence-based instructional design and likely rely on MI theory.

Multiple studies have provided evidence of MI theory’s semantic effectiveness in inspiring differentiated teaching and learning in the classroom (e.g., Modirkhamene and Azhiri, 2012; Milić and Simeunović, 2017; Koçak Altundağ, 2018; Ghaznavi et al., 2021; Shahzada et al., 2021). Semantic effectiveness here means that MI theory’s use of the word intelligences seemed to be interpreted as nearly synonymous or at least overlapping with preferences; therefore, its conceptual framing interfaced readily with learning styles theory. As such, qualitative observations tended to provide positive interpretations balancing somewhat ambivalent quantitative findings, depending on the study’s research design. Part of the issue in discussion of findings depends in part on how the word *intelligences* is used when considering the historically technical term of general intelligence (*g*) with the way in which it is pluralized and adapted in MI theory. Armstrong’s (2018) textbook on MI theory in the classroom further highlights its popularity and, as such, it might be noted in educational psychology courses to preservice teachers from a perspective of a way to think about differentiation. However, UbD and UDL should be taught throughout to emphasize these frameworks’ importance.

Teacher educators should refer to Gardner’s (1983, 2006) MI theory focusing on its qualitative value for curriculum design to foster student engagement in the content area. This could be a three-step process for the teacher educator: (1) emphasize that MI theory is a way to envision differentiation in designing projects that give students options, while noting the problem of validity when calling preferences “intelligences”; (2) implement activities in which preservice teachers practice project design in their content areas to address preferences when practicable through UbD or UDL; and (3) ask preservice teachers to think of MI theory through the lens of preferences in which each of the eight intelligences are preferences. It should also be noted that just because a student indicates that they prefer to learn a certain way, does not necessarily mean the student learns best that way (Papadatou-Pastou et al., 2018). This process should occur after teaching about the concept of general intelligence (*g*) and validity in psychological science since those concepts relate to standardized tests (Kane and Brand, 2006). Standardized tests are important for determining general intelligence historically as well as for establishing what services may be needed in special education, but are rarely part of the daily instructional side of student engagement as standardized tests tend to rely on the older concept of *g*. The increasing trend of colleges and universities discontinuing requirements of standardized tests such as the SAT and ACT for admission is an indication of changing perceptions or pressures on the role of a singular, general intelligence measure (*g*) when student eligibility for admission to universities or academic programs is under consideration (Sternberg, 2020; Vigdor and Diaz, 2020). Given this trend, MI theory may continue to be relevant in the broader discussion of the role in personalized learning and assessment.

Preservice teachers should be encouraged to learn about student engagement strategies which MI theory provides. MI theory can establish the foundation for engagement with the think-pair-share format of learning and differentiated curriculum design so that students think about the concept of general intelligence and then MI theory’s different approach to intelligence as preferences. In this way, preservice teachers are introduced to the technical aspects of how educational psychologists have defined intelligence while also engaging with MI theory in ways that are practicable for differentiation in the K-12 classroom.

In a unit on learning styles theories in preservice teachers’ educational psychology course, include a lesson on the precision of language and semantics as it relates to MI theory and disciplinary vocabulary. While MI theory is separate from learning styles theory, as it does differ, there is some thematic overlap in conceptual goals between the two (Silver et al., 1997). Engaging preservice teachers in an intentional discussion about the history of MI theory, its influence, and its promising concepts for fostering differentiation can be an integral part of having an informed opinion and understanding of MI theory. Explain the benefits of explicit direct instruction (see Hollingsworth and Ybarra, 2018) and its uses in teaching skills and content within UDL and UbD frameworks. Then, assign a project design assignment in which preservice teachers design a UDL or UbD lesson plan. Concurrently assign a lesson plan in which students are tasked with applying eight of the intelligences from MI theory to a differentiated project in their content area. Assign students into groups based on content area and grade level and have them discuss their completed project designs to compare across the frameworks.

## Conclusion

This review of the literature on the perceptions of MI theory and the related concept of learning styles theory suggests a continued divergence in observations of MI theory’s efficacy in relation to generalizable validity. Nevertheless, there are studies discussed in in this conceptual analysis that have showed favorable outcomes. Various studies discussed in this conceptual analysis align with findings from Shearer (2020) in the benefits of MI theory observed in those case studies. Some scholars in the literature reviewed also posited questions and points of view in this debate whose discussions should be included in courses on educational psychology for preservice teachers so that they have an informed view of the conceptual background of the theory throughout time. They may likely encounter MI theory or learning styles theory in their schools, so an informed view is important. A discussion of the semantics of the word intelligence with the concept of general intelligence (*g*) and its relationship to IQ should be included for context. The studies critical of MI theory as well as those that have had favorable observations should perhaps both be presented and discussed with preservice teachers, while UbD (see Wiggins and McTighe, 2005) and UDL (see Hall et al., 2012) should be emphasized as evidence-based frameworks for their K-12 classroom contexts.

## Author Contributions

The author confirms being the sole contributor of this work and has approved it for publication.

## Conflict of Interest

The author declares that the research was conducted in the absence of any commercial or financial relationships that could be construed as a potential conflict of interest.

## Publisher’s Note

All claims expressed in this article are solely those of the authors and do not necessarily represent those of their affiliated organizations, or those of the publisher, the editors and the reviewers. Any product that may be evaluated in this article, or claim that may be made by its manufacturer, is not guaranteed or endorsed by the publisher.
